# Content Analysis of Primary and Secondary School Textbooks Regarding Malaria Control: A Multi-Country Study

**DOI:** 10.1371/journal.pone.0036629

**Published:** 2012-05-04

**Authors:** Daisuke Nonaka, Masamine Jimba, Tetsuya Mizoue, Jun Kobayashi, Junko Yasuoka, Irene Ayi, Achini C. Jayatilleke, Sabina Shrestha, Kimiyo Kikuchi, Syed E. Haque, Siyan Yi

**Affiliations:** 1 Department of Parasitology and International Health, Graduate School of Medicine, University of the Ryukyus, Okinawa, Japan; 2 Department of Community and Global Health, Graduate School of Medicine, The University of Tokyo, Tokyo, Japan; 3 Department of Epidemiology and Prevention, International Clinical Research Center, National Center for Global Health and Medicine, Tokyo, Japan; 4 Graduate School of International Health Development, Nagasaki University, Nagasaki, Japan; 5 Department of International Medical Cooperation, National Center for Global Health and Medicine, Tokyo, Japan; 6 Parasitology Department, Noguchi Memorial Institute for Medical Research, University of Ghana, Accra, Ghana; 7 The Walter H. Shorenstein Asia-Pacific Research Center, Freeman Spogli Institute of International Studies, Stanford University, Palo Alto, California, United States of America; Kenya Medical Research Institute – Wellcome Trust Research Programme, Kenya

## Abstract

**Background:**

In tropical settings, malaria education at school is potentially useful, but textbook content related to malaria education has so far received little attention. This study aimed to examine whether school textbooks contain sufficient knowledge and skills to help children in primary and lower secondary schools and their family members to cope with malaria.

**Methodology/Principal Findings:**

This was a descriptive, cross-country study. We collected textbooks that were used by children in grades one to nine from nine countries endemic for malaria: Laos, Cambodia, Nepal, Bangladesh, Sri Lanka, Zambia, Niger, Benin, and Ghana. Two reviewers per country identified descriptions about malaria by seeking the term “malaria” or a local word that corresponds to malaria in languages other than English. The authors categorized the identified descriptions according to the content of the descriptions. Additionally, the authors examined whether the identified contents addressed life skill messages. Of a total of 474 textbooks collected, 35 contained descriptions about malaria. The most commonly included content was transmission mode/vector (77.1%), followed by preventive measures (60.0%), epidemiology (57.1%), cause/agent (54.3%), signs/symptoms (37.1%) and treatment (22.9%). Treatment-related content was not included in any textbooks from four countries and textbooks failed to recommend the use of insecticide-treated bed nets in five countries. Very few textbooks included content that facilitated prompt treatment, protection of risk groups, and use of recommended therapy.

**Conclusion/Significance:**

Textbooks rarely included knowledge and skills that are crucial to protect schoolchildren and their families from malaria. This study identified the need for improvement to textbook contents regarding malaria.

## Introduction

In many countries with endemic malaria, the general community does not necessarily know much about the disease. For example, it has been reported that many rural community members in Nigeria, Ethiopia and Bangladesh do not know how malaria is transmitted [1]–[3]. Such poor knowledge is often associated with inappropriate preventive behavior among members of the local community, and consequently can lead to increased risk of infection; people who understand little about the linkage between malaria and mosquito bites were more likely not to use a bed net in Ghana, Tanzania and India [4]–[6]. Moreover, people who did not know about the cause of malaria were more likely to have malaria in Tanzania and Zimbabwe [5], [7]. Lack of knowledge about symptoms and treatment of malaria can cause delays in seeking health care from trained health workers who usually diagnose malaria using microscopy to detect malaria parasites or by using a rapid diagnostic test kit [8].

There are few reports that have considered how knowledge about malaria control measures changes over time within a country or how this understanding varies between countries. However, a number of studies have suggested that urban dwellers are more likely to have greater knowledge of malaria than people who reside in rural areas because they have higher levels of literacy and greater exposure to educational media [9], [10].

Recently, researchers and international agencies have increasingly recognized the importance of malaria education targeted to schoolchildren [11]–[13]. Although the burden of malaria among children of school-going age has been largely overshadowed by the huge burden among young children, a recent review has shown that in unstable transmission settings, teenagers (10–19 years) encounter clinical episodes as often as young children, or sometimes more frequently. Globally, malaria is a common cause of death in adolescence, accounting for 7.4% of deaths from all causes [14]. Malaria also widely contributes to school absenteeism and poor academic achievement, accounting for 3–8% of all reasons for absenteeism [11], [13]. Thus, schoolchildren should be educated to cope with malaria. Additionally, schoolchildren are not merely recipients of malaria education but also can be health change agents; children can convey the knowledge and skills that they acquire at school to the community, thus increasing general community awareness about malaria [14], [15].

Schools have the potential to reach large portions of the population in an effective and efficient manner and play an important role in disseminating information to children and their families in rural communities where access to information via mass media, literature and the Internet is likely limited. For example, a study conducted with schoolchildren in Tanzania reported that the primary source of information about malaria was the school/teachers [16].

A number of studies have analyzed the content of textbooks used in primary and/or secondary schools to assess health education messages delivered at school [17]. However, these studies focused chiefly on sexually transmitted diseases, reproductive health and nutrition issues [17] and, to the best of our knowledge, no published data are available regarding malaria in school textbooks.

This study aimed to examine whether textbooks in malaria-endemic countries contain sufficient knowledge and skills to help schoolchildren and their family members to cope with malaria.

## Methods

### Textbook collection

This was a descriptive, cross-country study. We collected a total of 474 textbooks that were used for primary and lower secondary school students from nine Asian and African countries where malaria still remains a threat to the population and where the authors have established relationships with local collaborators. These countries (number of collected textbooks) were Laos (63), Cambodia (37), Nepal (69), Bangladesh (70), Sri Lanka (68), Zambia (41), Niger (57), Benin (36) and Ghana (33).

We collected information about textbooks from the education sector such as the Ministry of Education and local education department in each country. Then we obtained textbooks from bookshops, the Ministry of Education, or through organizations that are authorized by the government to distribute textbooks to schools. These textbooks covered all subjects taught in public schools from grades one to nine. As the types of textbooks used in public schools differ according to the school, the textbooks that were most commonly used were collected. Textbook collection took place between January 2009 and March 2010.

### Textbook examination

In each county, two public health experts reviewed the contents of the textbooks and identified descriptions about malaria by seeking the term “malaria” or a term that corresponded to malaria in a language other than English. Non-English descriptions were translated into English. The textbooks of Ghana and Zambia, and some textbooks for foreign language subjects were written in English. The second reviewer checked the results of the first reviewer. When there was a discrepancy between the two reviewers on identification of descriptions or translation results, they re-examined the material for a solution.

### Content analysis

The authors categorized the identified descriptions of the textbooks according to the content of the descriptions. The content categories included transmission mode/vector, cause/agent, signs/symptoms, treatment, preventive measures and epidemiology.

Additionally, the authors examined whether the identified descriptions address the key messages presented in Facts for Life, which is a book published by the United Nations and designed to deliver life skills information on how to prevent child and maternal deaths, injuries and violence [18]. This book has been used elsewhere to assess health knowledge among women in rural communities [19], [20]. Although there are four key messages pertaining to malaria in this book, three of these key messages were comprised of multiple sentences and were difficult to use for assessment in this study in its original form, so they were further divided into six to make a total of seven key messages.

## Results

### Characteristics of textbooks

Of a total of 474 textbooks, 35 (7.4%) textbooks contained descriptions about malaria. Most of these textbooks were designed for science-related subjects whereas four textbooks for language subjects also referred to malaria in Zambia and Niger (Table 1).

**Table 1:** Subjects and grades of school textbooks containing content pertaining to malaria.

	Content						
a	Transmission mode/vector	Cause/agent	Signs/symptoms	Treatment	Preventive measures	Epidemiology	Other
1	World around us (4)	World around us (4)	World around us (4)	World around us (4)	World around us (4)	World around us (4)	Science (8)
	Science (8)	Science (8)	Science (8)	Science (8)	Science (8)	Science (8)	
2	Practical science (5)	Practical science (5)	Practical science (5)	Practical science (5)	Practical science (5)	Practical science (5)	
	Sociology (9)	Sociology (9)	Sociology (9)	Sociology (9)	Sociology (9)	Sociology (9)	
3	Science (4)	Science (4)	Science (4)		Science (4)		
	Home economics (8)				Home economics (8)		
4	Health & physical education (6)	Health & physical education (6)	Health & physical education (6)	Health & physical education (6)	Health & physical education (6)	Health & physical education (6)	
5	Health & physical education (7)	Science (8)	Health & physical education (7)		Health & physical education (7)	Science (8)	Science (8)
	Science (8)				Science (8)		
6	Social & development studies (2)	Integrated science (3)	Creative & technology studies (6)		Social & development studies (2, 7)	Social & development studies (2, 7)	English (2)
	Integrated science (3)	Environmental science (5)			Environmental science (5)	Integrated science (3, 7)	Social & development studies (7)
	Environmental science (5)	Creative & technology studies (6)			Integrated science (7)	English (4,7)	
	Creative & technology studies (6)	English (9)			English (7)	Creative & technology studies (6)	
	English (7, 9)					Environmental science (8)	
7	Reading & writing (2, 5)	Science (5)	Science (5)	Reading & writing (5)	Reading & writing (5)	Reading & writing (2)	Reading & writing (2)
	Science (5)	Biology (7, 9)	Biology (9)	Science (5)	Science (5)	Science (5)	
	Biology (7, 9)					Biology (9)	
8	Science (4–5)	Life & earth science (7)	Science (4–5)		Science (4–5)	Life & earth science (7)	
	Life & earth science (7)	Biology (7, 9)	Biology (9)		Life & earth science (7)	Biology (9)	
	Biology (7, 9)						
9	Social studies (5)	Integrated science (8, 9)	Integrated science (9)	Integrated science (9)	Citizenship education (4,5)	Integrated science (9)	Citizenship education (4)
	Citizenship education (5)				Integrated science (9)		Social studies (9)
	Integrated science (9)						Integrated science (9)

a : Country ( 1: Laos, 2: Cambodia, 3: Bangladesh, 4: Nepal, 5: Sri Lanka, 6: Zambia, 7: Niger, 8: Benin, 9: Ghana)

The number of textbooks that referred to malaria was higher in African countries than in Asian countries: one or two textbooks per country in Asian countries whereas four or more in African countries.

The grade at which malaria education was initiated varied from country to country. The descriptions about malaria first appeared in grade two textbooks in Niger and Zambia, grade four in Bangladesh, Laos, Ghana and Benin, and grade five or higher in Cambodia, Nepal and Sri Lanka.

In every county, textbooks consistently covered content regarding transmission mode/vector, cause/agent, signs/symptoms and preventive measures. However, none of the textbooks used in Bangladesh, Sri Lanka, Zambia and Benin contained treatment-related content.

Most of the textbooks (28, 80.0%) were published in the target countries and all of the textbooks used in Asian target countries (9) were published by a government body. The initial years of publication raged from 1990 to 2009; nine textbooks have been revised after publication. Niger's biology textbooks for grade seven and nine were the same as those of Benin (Table S1).

### Characteristics of the content

Of the 35 textbooks that referred to malaria, more than half of these contained information regarding transmission mode/vector (27, 77.1%), cause/agent (19, 54.3%), preventive measure (21, 60.0%) and epidemiology (20, 57.1%). Less than half of the textbooks contained content regarding signs/symptoms (13, 37.1%) and treatment (8, 22.9%).

Although most of the textbooks that referred to malaria included information about transmission mode/vector, these textbooks did not necessarily specify anopheles mosquitoes as the vector: 14 textbooks (40.0%) mentioned that mosquitoes can transmit malaria, without specifying the anopheles mosquito, whereas 13 (37.1%) mentioned the transmission mode, specifying anopheles (Table 2). Similarly, 11 textbooks (31.4%) mentioned that a parasite or germ is the cause/agent, whereas 8 (22.9%) named the cause/agent, specifying plasmodium. Whenever textbooks referred to signs/symptoms, fever was presented together with one or more other symptoms. Only 4 textbooks (11.4%) presented information about danger signs such as convulsions and unconsciousness. When textbooks referred to treatment, they often introduced therapies other than artemisinin-based combination therapy: 4 textbooks (11.4%) presented information about treatments such as quinine, chloroquine or herbal medicines, whereas only one textbook introduced artemisinin-based combination therapy. Textbooks rarely included information about the danger of inappropriate treatment (11.4%), the need to seek care from a health worker (8.6%) and need for prompt treatment (8.6%).

**Table 2:** Details of content pertaining to malaria in textbooks.

Content	n (n = 35)	%
Transmission mode/vector		
Mosquito	14	40.0
Anopheles mosquito	13	37.1
Breeding site	9	25.7
Other	9	25.7
Cause/agent		
Plasmodium	11	31.4
Parasite/germ/poison	8	22.9
Incubation period	4	11.4
Other	2	5.7
Signs/symptoms		
Fever and other symptoms	13	37.1
Recurrent fever involving coldness and sweating	8	22.9
Danger signs	4	11.4
Treatment		
Therapy other than artemisinin-based combination therapy	4	11.4
Danger of inappropriate treatment and/or treatment delay	4	11.4
Need to seek care from health worker	3	8.6
Need for prompt treatment	3	8.6
Artemisinin-based combination therapy	1	2.9
Need to follow-up patients	1	2.9
Need for adhering to prescriptions	1	2.9
Prevention of dehydration and malnutrition	1	2.9
Preventive treatment during pregnancy	1	2.9
Other	2	5.7
Prevention		
Bed nets	14	40.0
Source control by environmental modification	14	40.0
Cleaning/weeding grass in and around house compounds	12	34.3
Mosquito coils and/or sprays	8	22.9
Source control by the use of chemicals	6	17.1
Chemo-prophylaxis	5	14.3
Source control by larvivorous fish	4	11.4
Repellents	3	8.6
Mosquito screen on windows	2	5.7
Other	5	14.3
Epidemiology		
Morbidity, mortality and characteristics of endemic regions	17	48.6
Consequence/adverse effect	16	45.7
Risk group (children and/or pregnant women)	5	14.3
Risk factor	2	5.7
Other		
Non-specific information, awareness creating message etc.	8	22.9

The most commonly included preventive measure was the use of bed nets (40.0%) and source control by environmental modification (40.0%), followed by cleaning/weeding grass in and around the house compound (34.3%) and use of mosquito coils/sprays (22.9%). Some textbooks also included epidemiological information such as morbidity and mortality due to malaria (48.6%) and the consequences/adverse effects of malaria (45.7%). Few textbooks (5.7%) presented information about risk groups such as children and pregnant women.

### Assessment with Facts for Life

In every country, at least one textbook had content that corresponded to the key message that “Malaria is transmitted through the bites of some mosquitoes” (Table 3). In the four countries of Laos, Cambodia, Zambia and Ghana, textbooks contained information that indicated the meaning of the key message that “Sleeping under an insecticide-treated bed net is a way to prevent mosquito bites.” Cambodia's textbooks addressed four other key messages of Facts for Life relating to children and pregnant women as the risk group, including the need for prompt treatment from a health worker, the need for preventive treatment during pregnancy and the need for plenty of liquid and food for children suffering or recovering from malaria. In addition, one textbook from Ghana introduced artemisinin-based combination therapy as one of the treatment choices. However, the textbook did not mention whether the treatment is recommended.

**Table 3:** Presence of textbook content that corresponds to key messages of Facts for Life.

Key messages of Facts for Life	Country^a^								
	1	2	3	4	5	6	7	8	9
Malaria is transmitted through the bites of some mosquitoes.	✓	✓	✓	✓	✓	✓	✓	✓	✓
Sleeping under an insecticide-treated bed net is a way to prevent mosquito bites.	✓	✓				✓			✓
Malaria is very dangerous for children and pregnant women.		✓							
Where malaria is present, a child with a fever should be examined immediately by a trained health worker.		✓							
Artemisinin-based combination therapies are recommended for treatment of *Plasmodium falciparum* malaria.									✓^b^
Wherever malaria is common, pregnant women should prevent malaria by taking antimalarial tables recommended by a trained health worker.		✓							
A child suffering or recovering from malaria needs plenty of liquids and food.		✓							

a : 1: Laos, 2: Cambodia, 3: Bangladesh, 4: Nepal, 5: Sri Lanka, 6: Zambia, 7: Niger, 8: Benin, 9: Ghana.

b : Insufficient description.

## Discussion

This study showed that textbooks provided information about transmission mode/vector, cause/agent and preventive measures of malaria in every country, whereas in four countries no information was provided about treatment. Textbooks often lacked life skills information including the use of an insecticide-treated bed net (ITN), the need for prompt treatment from a health worker, the protection of children and pregnant women from malaria, the recommended therapy, and the prevention of dehydration and malnutrition due to malaria.

The findings suggest a need for improvement of school textbook content pertaining to malaria. In Kenya and Uganda, for example, children often practiced self-treatment of their malaria-like illnesses or provided their younger siblings with treatment in an inappropriate manner [21], [22]. Due to such risky child behavior, researchers and international agencies have emphasized the importance of educating schoolchildren about life skills to cope with malaria, within the framework of the existing curricula [11]–[14], [23], [24].

In response to a remarkable increase in the school enrolment rate and the availability of textbooks at schools in tropical and sub-tropical countries [25], the importance of textbooks has been increasing. For example, in the nine target countries there are 35 million children that make up the primary school-age population, and potentially most of them use the textbooks that were examined in this study. Therefore, improvement to the content of school textbook could have a large impact on community knowledge and skills to fight against malaria.

Although ITNs have been adopted as a national malaria control strategy in all the target countries [26], the results here have shown that ITNs were not discussed in any textbooks in five countries. Use of an ITN is the best way to prevent mosquito bites in terms of cost-effectiveness and effectiveness in reducing morbidity and mortality [27], [28]. However, a number of studies have reported that many people do not use an ITN because their benefits are poorly understood [6], [29]–[31]. Incorporation of information regarding ITNs into textbooks has the potential to help promote their proper use in communities.

Our results showed that only one textbook in Cambodia presented information about the need for taking recommended antimalarial tablets by pregnant women; i.e., preventive treatment during pregnancy. Inclusion of information regarding preventive treatment may not be relevant in textbooks used in the Asian target countries, as preventive treatment is not recommended in such a low endemic setting [26]. Except for this point, however, all of the key messages of Facts for Life must be commonly relevant to the target countries and should be discussed in textbooks.

Our results also showed that content addressing treatment and symptoms was least likely to be included in the textbooks. Additionally, textbooks in four countries lacked treatment-related content. Generally, health education in school curricula tends to focus on biomedical knowledge and prevention, but not on actively coping with illness, as treatment practices are considered the role of medical experts [21]. However, as treatment for malaria should start within 24 hours of the onset of symptoms [32], children should be equipped with knowledge and skills to recognize symptoms of malaria and to make an appropriate treatment choice [23].

Even when textbooks contained treatment-related content, textbooks often introduced therapies other than artemisinin-based combination therapy, which has been adopted as the first line treatment for uncomplicated falciparum malaria in the target countries. This indicates the gap between therapies introduced in textbooks and national malaria control strategies. This gap might be due partly to infrequent updating of textbook information, as chloroquine which was previously the first line treatment in many countries was most commonly discussed in textbooks.

As Table S1 shows, the textbooks examined were published or revised between 1990 and 2010 (median year: 2006). In the target countries, ITNs and artemisinin-based combination therapy have been adopted as a national malaria control strategy, between 1992 and 2000 and between 2000 and 2008, respectively. Especially in the African target countries, preventive treatment during pregnancy was also adopted between 2001 and 2005 [26], [33], [34]. Hence, textbooks that were published or revised before these periods are likely to lack content related to these strategies. In contrast, general scientific knowledge about malaria (e.g., risk group, the need for prompt treatment and the prevention of dehydration and malnutrition as shown in Table 3) have not changed over time, and thus the absence of such information from textbooks may not be due to infrequent updating of textbooks, but possibly due to insufficient collaboration between the education and health sectors during textbook development.

As also shown in Table S1, many textbooks had not been revised frequently; 15 out of 35 textbooks had not been revised for more than five years since their publication or previous revision. Resource-rich countries can overcome infrequent updating by adopting information communication technologies. Electronic textbooks enable publishers to easily update content when content becomes out-of-date [35]. For example, more than 600 school districts in the United States have adopted an iPad program without paper textbooks [36]. Although, due to infrastructure constraints, replacing paper textbooks with electronic ones is currently unlikely to be realistic in many resource-limited countries, including those in our study, electronic textbooks or other electronic media may play an important role in the future in providing schoolchildren with up-to-date information.

In contrast to the wide gap between textbook content and most of the key messages of Facts for Life, the results showed that in every country the key message that “malaria is transmitted through the bites of some mosquitoes” was introduced in the textbooks. One concern is whether the textbook content actually translates into knowledge among children. A study conducted with primary school children in Ghana, where the present study found that textbooks included information regarding transmission mode and ITNs, reported that the participating children had a good level of knowledge; 76.2% (95% confidence interval: 67.9–84.5) of grade three to five primary school children knew how malaria is transmitted and 80.4% (72.2–87.8) knew about ITNs [15]. In contrast, 64.0% (56.7–71.2) of grade three primary school children did not know how malaria is transmitted in Zimbabwe where malaria information is taught at grade four or higher [7].

As Table 3 shows, Cambodia's textbooks had the most complete presentation of life skills information among the defined Facts for Life, whereas textbooks in other countries failed to present most of this information. In Cambodia the School Health Policy advocates integration of life skills education on nutrition, hygiene, and disease prevention into the school curriculum [37], and the Ministry of Health is involved in curriculum development [38]. These school health efforts might be attributable to the comprehensiveness of life skills information in Cambodia's textbooks. In Cambodia, the number of malaria cases has decreased slightly at the national level and in some parts of the country decreased remarkably, by more than 50%, between 2000 and 2010. Although the roles of village malaria workers seem to be critical [39], improved textbooks might be one of the factors associated with the decreasing malaria trends in this country.

Another clear difference between countries is that textbooks of the African target countries were more likely to refer to malaria than those of Asian target countries. This difference might reflect the higher burden of malaria in African children.

According to the United Nations Educational, Scientific and Cultural Organization (UNESCO), primary school children are expected to learn about mosquito breeding places in lower grades, transmission, cause and prevention in middle grades and means to care for people who are sick with malaria in higher grades [24]. The results of this study indicated that in most of the studied countries malaria health education started with grade four or higher, suggesting a possible need for earlier initiation of malaria health education in the target countries.

This study focused on textbooks alone. However, textbooks are not the sole learning materials in classrooms; electronic learning media are increasingly used, particularly in resource-rich countries [36] and even in some of the resource-limited countries included in our target area, paper-based learning materials other than textbooks can sometimes be available. For example, in Laos, “Blue Box”, which is a package of health education materials including a storybook for malaria, has been developed with support from international agencies and has been made available at many primary schools. Compared to textbooks, however, the supply of such extra learning materials is more likely to be susceptible to reduced external funding in resource limited settings. Additionally, extra materials are unlikely to be sustainable if they are not integrated into the curricula [40].

### Limitations

This study has four major limitations. First, the sampling was confined to textbooks used for children up to grade nine. As there may be malaria-related content in textbooks for children in higher grades and in learning materials other than textbooks for all grades, the results of this study do not entirely reflect the malaria education programs in the target countries. Second, although two reviewers were involved in the identification of descriptions of malaria in each target country, the review was not done independently. Thus, the results of this study might be biased compared to those produced by independent reviews. Third, this study only adopted descriptions that were obviously linked with malaria. For example, general descriptions about nuisance control, maintenance of a clean environment or first-aid were not included. Therefore, the results of the present study could underestimate descriptions that are indirectly connected with malaria control. Finally, the accuracy of the information provided in textbooks was not taken into account. For example, de Irala *et al.* reported that the content of Spanish science textbook regarding sexually transmitted diseases and reproductive health sometimes provided inaccurate information [41]. Further study is necessary to assess the accuracy of content pertaining to malaria in school textbooks.

### Conclusion

The textbooks examined in this study rarely addressed life skills information such as the use of ITNs, the need for prompt treatment from a health worker, protection of children and pregnant women, recommended therapy, or the prevention of dehydration and malnutrition due to malaria. This suggests that the current generation of textbooks plays a very limited role in conveying knowledge and skills that help schoolchildren and their family members cope with malaria in the countries in this study. Improving school textbook content in accordance with a national malaria control strategy could be the key challenge in malaria endemic countries to increase children's access to life skills information.

## Supporting Information

Table S1 Textbook publishers, authors, and years of publication/revision.
(DOC)
