# Supplementary material for: Content Analysis of Primary and Secondary School Textbooks Regarding Malaria Control: A Multi-Country Study
Source: PLoS One. 2012 May 4;7(5):e36629. doi: 10.1371/journal.pone.0036629 (PMC3344925; doi:10.1371/journal.pone.0036629)
Supplement: Table S1 — Textbook publishers, authors, and years of publication/revision. (DOC) [file pone.0036629.s001.doc]

**Table S1.** Textbook publishers, authors and years of publication/revision.

| **Country** | **Textbook (Grade)** | **Publisher** | **Publisher location** | **Authors** | **Published　year** | **Revised year** |
| --- | --- | --- | --- | --- | --- | --- |
| Laos | Science (8) | Ministry of Education, Laos | Vientiane, Laos | Khambounphan P, Sibounhueang P, Nanthavoung B, Khounphilaphan B, Keosada H, Silalak B, Philavong V | 1997 | - |
|  | World around us (4) | Ministry of Education, Laos | Vientiane, Laos | Xaynyavong B, Wainyakoun S | 2009 | - |
| Cambodia | Practical science (5) | Ministry of Education, Youth and Sport | Phnom Penh, Cambodia | Kimsan I, Thysaron T, Sary K, Prasoeu T, Hak S, Hak K, Srong T, An SP | 2000 | 2005 |
|  | Sociology (9) | Ministry of Education, Youth and Sport | Phnom Penh, Cambodia | Yahon C, Pao P, Kimsen B, Bunheng N, Yar K, Chutema M, Nim P, Somaly N, Neang B, Vanthany M, Sokhema T, My L | 1999 | - |
| Bangladesh | Science (4) | National Education and Textbook Board | Dhaka, Bangladesh | Ministry of Primary and Mass Education | 2004 | - |
|  | Home economics (8) | National Education and Textbook Board | Dhaka, Bangladesh | Ministry of Education | 1996 | - |
| Nepal | Health and physical education (6) | Ministry of Education,  Curriculum Development Center | Bhaktapur, Nepal | Maharjan RK, Shrestha HP, Serchan L, Maharjan SK, Rokaya RB | 1994 | 2001 |
| Sri Lanka | Health and physical education (7) | Educational Publications Department | Colombo, Sri Lanka | Pushpakumara WMNJ, Nalika WDP, Piyaseeli WAN, Abeywickram A, Senanayak S, Piyas WAN, Sugathadas S | 2007 | - |
|  | Science (8) | Educational Publications Department | Colombo, Sri Lanka | Pushpakumara WMNJ, Nanayankkara EFD, Nalika WDP, Ranasinghe RAD, Sriyalatha KVN, Kumara KDB, Ratnatilaka AAL, Silva ADAD, Ranatunga S, Ariyasingha K, Siriwardhana AWA, Jayaratne HTCG, Disanayaka LADIS, Adamlebbe J | 2008 | - |
| Zambia | English (2) | Cambridge University Press | Cape Town, South Africa | Londt C, Morrison K, Tonkin S | 2006 | - |
|  | English (4) | Longman Zambia Ltd. | Lusaka, Zambia | Chisense W, Gondwe ES, Mupinde C,  Mundambo LM, Shipota M | 2007 | - |
|  | English (7) | Longman Zambia Ltd. | Lusaka, Zambia | Chisense W, Gondwe ES, Mupinde C,  Mundambo LM, Shipota M | 2007 | - |
|  | English (9) | Macmillan Publishers Zambia Ltd | Lusaka, Zambia | Hurry B, Zimba S | 2003 | - |
|  | Environmental science (5) | Juta & Co. Ltd. | Cape Town, South Africa | Mashambe CM, Lupele JK, Hamooya BM,  Shiyanda J | 1996 | - |
|  | Environmental science (8) | Zambia Educational Publishing House | Lusaka, Zambia | Banda AJ, Mudenda VJ, Chengo A, Tindi E,  M'hango IY, Ziwa AJ | 1990 | 1994 |
|  | Social and development studies (2) | Maiden Publishing House | Lusaka, Zambia | Mushiko ECB, Zulu S, Tembo CM | 2005 | - |
|  | Social and development studies (7) | Longman Zambia Ltd. | Lusaka, Zambia | Musonda M, Simasiku S, Mayondi C | 2007 | - |
|  | Integrated science (3) | Longman Zambia Ltd. | Lusaka, Zambia | Chisala FC, Phiri D, Sakala E, Shampile L | 2006 | - |
|  | Integrated science (7) | Longman Zambia Ltd. | Lusaka, Zambia | Chisala F, Phiri DS, Shampile L, Sikapizye A | 2007 | - |
|  | Creative and technology studies (6) | Cambridge University Press | Cape Town, South Africa | Wakumelo M | 2006 | - |
| Niger | Science (5) | National Institute for Pedagogical Documentations Research and Promotion | Niamey, Niger | Adamou A, Arouna M, Biba M, Gaoh DRne (Editor) | 2009 | - |
|  | Reading and writing (2) | National Institute for Pedagogical Documentations Research and Promotion | Niamey, Niger | Dembel DA, Abba M, Tinga D, Semandi O, Hamadou M, Foumakoye AN | 2009 | - |
|  | Reading and writing (5) | National Institute for Pedagogical Documentations Research and Promotion | Niamey, Niger | Dembel DA, Maїromēya H, Maїnassara C,  ouOusmane S | 1994 | - |
|  | Biology (7) | Bordas | Paris, France | Djakou R, ouThanon YS | 1991 | 1996 |
|  | Biology (9) | Bordas | Paris, France | Djakou R, ouThanon YS | 1991 | 1996 |
| Benin | Science (4-5) | Nathan | Paris, France | Not indicated | 1999 | - |
|  | Life and earth science (7) | Hatier International | Paris, France | Panaf | 2002 | - |
|  | Biology (7) | Bordas | Paris, France | Djakou R, ouThanon YS | 1991 | 1996 |
|  | Biology (9) | Bordas | Paris, France | Djakou R, ouThanon YS | 1991 | 1996 |
| Ghana | Citizenship education (4) | Unimax Macmillan Ltd. | Accra, Ghana | Otu C, Quartey SM, Skelt J | 2008 | - |
|  | Citizenship education (5) | Unimax Macmillan Ltd. | Accra, Ghana | Otu C, Quartey SM, Skelt J | 2008 | - |
|  | Social studies (5) | EPP Books Services | Accra, Ghana | Nkansah B, Palm H, Awuku A, Tahil M, Benkyi GO, Fomena A | Not indicated | 2010 |
|  | Social studies (9) | Adwinsa Publications Ltd. | Accra, Ghana | Amoah EA, Baabereyir A, Cobbinah JA, Dake GY, Ngaaso CK | 2005 | 2008 |
|  | Integrated science (8) | Sedco-Longman Sedco Publishing Ltd. | Accra, Ghana | Wiredu MB, Doku T, Joe-Adjei EC | 2008 | - |
|  | Integrated science (9) | Sedco-Longman Sedco Publishing Ltd. | Accra, Ghana | Wiredu MB, Doku T, Joe-Adjei EC | 2008 | - |
